# Exploring Australian Women’s Attitudes Towards Paid Menstrual Leave: A Mixed‐Methods Study

**DOI:** 10.1111/ajo.70031

**Published:** 2025-03-31

**Authors:** Amy Wong, Melissa Oxlad, Deborah Turnbull

**Affiliations:** ^1^ School of Psychology The University of Adelaide Adelaide South Australia Australia

**Keywords:** Australia, menstrual health, menstruation, paid menstrual leave, workplace accommodations

## Abstract

**Background:**

Australian lawyers, researchers and unions are advocating for the implementation of national paid menstrual leave legislation. The scarce research in this emerging field has not thoroughly explored women's attitudes towards such leave.

**Aim:**

To explore Australian women's attitudes towards paid menstrual leave.

**Materials and Methods:**

Participants (*n* = 923, female sex assigned at birth currently or previously menstruating; 18+ years; resident in Australia; fluent in English) primarily recruited via social media, including accounts of Australian women's health and menstruation support groups for conditions such as adenomyosis and endometriosis, using a passive snowball sampling frame, completed an online cross‐sectional mixed‐methods survey.

**Results:**

Most (85%) expressed being somewhat to definitely in favour of paid menstrual leave, 4% were unsure and 11% were somewhat to definitely not in favour. Younger age and history of missing work due to menstrual pain were significant independent predictors of support for paid menstrual leave. Women preferred menstrual leave to be paid (rather than unpaid or reimbursed as additional income if unused), available monthly, with a once‐off doctor's approval required to access leave. Overall, 73% of participants reported positive impacts on employees in response to open‐ended questions about potential impacts (positive, negative and unintended). The most common categories were better workplaces and productivity and improved work outcomes.

**Conclusion:**

Research such as this is essential for informing considerations about menstrual leave legislation. Further research investigating the attitudes of a wider range of stakeholders, such as men, colleagues, employers, employment lawyers and economists, is needed to develop an inclusive understanding of menstrual leave.

## 1 Introduction

Menstrual health is a public health issue concerned with one's physical, mental and social wellbeing related to the menstrual cycle [1]. While menstruation is a normal health function, menstrual‐related pain is one of the most common causes of pain for women of reproductive age [2]. A recent meta‐analysis of over 21,000 young women indicated that 90% of respondents reported debilitating pain during their periods, with 40% of those respondents missing study and work commitments to cope with or hide their pain [3]. Menstrual‐related pain is more common and severe for women with health conditions such as dysmenorrhea and endometriosis [4]. Research indicates that women often use sick leave for menstrual‐related symptoms and that among those with health conditions, such as endometriosis, it is common to exhaust sick and unpaid leave for symptoms related to these health conditions and menstruation [5, 6].

Paid menstrual leave is a type of employment leave available to those whose menstrual‐related symptoms prevent them from working safely and productively [7]. Australian lawyers, researchers and unions have been advocating for the implementation of national paid menstrual leave legislation. They have argued that paid menstrual leave would put women on an equal footing with their colleagues and could be a potential solution to protect women's health and safety [7, 8]. In Australia, individual organisations such as *Future Super*, *Modibodi* and the *Victorian Women's Trust* have enacted menstrual leave policies [9]. However, the limited peer‐reviewed research in this emerging field has not thoroughly explored Australian women's attitudes towards such leave. Existing studies on menstrual leave in countries such as America have reported various perceived effects of paid menstrual leave. For example, benefits such as protecting women who miss work during menstruation to concerns of potential stigmatisation and violation of women's privacy, which may lead to discrimination [4].

To contribute to a comprehensive understanding about how menstrual leave might be received in Australia, we investigated Australian women's (i) levels of support for paid menstrual leave, (ii) preferences for paid menstrual leave and other menstruation‐related workplace accommodations, (iii) predictors of support for paid menstrual leave and (iv) attitudes towards the potential positive, negative and unintended impacts of paid menstrual leave on employees.

## 2 Materials and Methods

### 2.1 Participants and Data Collection

We employed a concurrent mixed‐methods design. Data were collected via an online cross‐sectional survey hosted on Qualtrics. The University of Adelaide School of Psychology Human Research Ethics Sub‐Committee approved the research (ethics approval number 2022‐78). To be eligible, participants were to be aged 18+ years, fluent in English, reside in Australia and have experience with menstruation (current or previous). We followed the Sex and Gender Equity in Research (SAGER) guidelines and employed its reporting checklist to shape the content of this paper [10]. All participants had to be female sex assigned at birth, regardless of their current gender, to ensure they had experience with menstruation.

Participant recruitment occurred predominantly via social media, including the researchers' accounts, and community groups, including a range of Australian women's health and menstruation support groups for adenomyosis, polycystic ovarian syndrome, endometriosis, premenstrual dysphoric disorder, pelvic pain and menopause. Other groups that shared study information and/or stories on social media included TABOO Period Products and The Chalice Foundation. Participants were also recruited via the Body@Work Project's mailing list, the first‐year psychology students' Research Participation System at the University of Adelaide (for course credit) and broader passive snowball sampling.

### 2.2 Measures

Participants completed a purpose‐designed survey comprising three sections: demographics (i.e., age, sex assigned at birth, gender, sexual orientation, country of birth, highest level of education completed, employment status, employment industry), menstruation history (i.e., current menstruation status, experience of debilitating period pain, formal diagnosis of painful periods by a health professional and if they ever had to skip work, cancel a shift or use leave for menstrual‐related pain) and attitudes towards paid menstrual leave (participants indicated, on a 5‐point Likert scale (*Definitely yes* to *Definitely no*), whether they were in favour of paid menstrual leave). Participants who supported paid menstrual leave proceeded to more detailed questions regarding other workplace accommodations for menstruation and menstrual leave preferences. All participants, regardless of their support for paid menstrual leave, were asked two open‐ended questions about the potential positive and negative or unintended impacts of paid menstrual leave on employees.

### 2.3 Data Analysis

Binary logistic regression was used to explore factors associated with support for paid menstrual leave (i.e., age, employment status, current menstrual status, experience of debilitating pain and history of missing work due to menstrual pain). The factors explored were selected a priori based on limited existing research and considerations of the research team [6, 11, 12]. All predictor variables were entered into the model simultaneously using the enter method [13].

Participants' responses to two open‐ended questions were analysed using classical inductive content analysis [14]. The first author familiarised themselves with the data through multiple readings, and then all researchers independently analysed 10 surveys [15]. Findings were discussed to validate and guide subsequent coding. After agreeing on the coding structure, all responses to the two questions were analysed separately, and responses of similar content were merged and assigned a code name. Two units of analysis were used: [1] the number of participants who provided textual responses relevant to each code and [2] the number of responses applicable to each code.

## 3 Results

Nine hundred and twenty‐three respondents participated; of the 724 participants who completed the demographic questions, 95% reported their current gender as female, with most being born in Australia (Table 1). Most participants were below 45 years of age, had at least completed high school and were currently employed or studying. Almost one‐half had a medical diagnosis of menstrual pain, and most reported missing work, cancelling a shift or using leave for menstruation‐related pain.

**TABLE 1:** Summary of participants' demographic and menstruation information.

Demographic characteristic	*N* (%)
Sex assigned at birth (*N* = 724)	
Female	724 (100%)
Current gender (*N* = 724)	
Female	695 (95.3%)
Male	4 (0.5%)
Non‐binary	17 (2.3%)
I/They use a different term	6 (0.8%)
Non‐binary woman (She/They)	2 (0.3%)
Agender	1 (0.1%)
Androgynous	1 (0.1%)
Demigirl	1 (0.1%)
Trans masc woman	1 (0.1%)
Sexual orientation (*N* = 724)	
Heterosexual	498 (68.8%)
Gay	23 (3.2%)
Bi+ (bisexual, pansexual)	160 (22.1%)
I use a different term	13 (1.8%)
Queer	4 (0.6%)
Demisexual	2 (0.3%)
Panromantic asexual	1 (0.1%)
Pansexual	1 (0.1%)
Don't know	16 (2.2%)
Prefer not to say	14 (1.9%)
Age (*N* = 721)	
18–24	304 (42.2%)
25–34	256 (35.5%)
35–44	95 (13.2%)
45–54	40 (5.6%)
55–64	26 (3.6%)
Country of birth (*N* = 724)	
Australia	591 (82.3%)
Other	129 (17.7%)
Education (*N* = 724)	
Less than year 12	13 (1.8%)
Completed year 12	144 (19.9%)
Vocational/Trade certificate	57 (7.9%)
Undergraduate university study	304 (42%)
Postgraduate university study	188 (26%)
Other	15 (2.1%)
Employment status (*N* = 742)	
Full time	269 (37.2%)
Part time	84 (11.6%)
Casual work	61 (8.4%)
Self employed	19 (2.6%)
Student (currently working)	197 (27.2%)
Student (not currently working)	75 (10.4%)
Retiree	7 (1.0%)
Unemployed	12 (1.7%)
Experience of debilitating menstrual pain (*N* = 721)	
Yes	609 (84.5%)
No	112 (15.5%)
Diagnosis of menstrual pain by health professional (*N* = 608)	
Yes	261 (42.9%)
Endometriosis	58 (22.2%)
Polycystic ovarian syndrome (PCOS)	38 (14.6%)
Dysmenorrhea	28 (10.7%)
Adenomyosis	26 (10.0%)
Fibroids	8 (3.1%)
Premenstrual dysphoric disorder (PMDD)	8 (3.1%)
Other	95 (36%)
No	347 (57.1%)
Frequency of skipping work, cancelling a shift or using leave for pain related to menstrual cycle (*N* = 720)	
Yes	
Frequently	152 (21.1%)
Occasionally	213 (29.6%)
Rarely	187 (26.0%)
No	168 (23.3%)

Most respondents (85%) were *Somewhat* to *Definitely* in favour of paid menstrual leave, with 4.3% *Definitely not* in favour and 4.4% *Unsure* (Table 2). Many participants would use menstrual leave if it were available (72% of those who currently menstruate would use it; 60% of those who no longer menstruate would have used it if it had been available to them).

**TABLE 2:** Preferences for menstrual leave and menstruation‐related workplace accommodations.

Preferences	*N* (%)	95% confidence interval	
		Lower	Upper
Levels of support for leave (*N* = 923)			
Definitely yes	619 (67.1%)	0.64	0.70
Somewhat yes	166 (18%)	0.16	0.21
Unsure	41 (4.4%)	0.04	0.07
Somewhat no	57 (6.2%)	0.05	0.08
Definitely no	40 (4.3%)	0.03	0.06
Financial preference (*N* = 623)			
Paid	524 (84.1%)		
Unpaid	25 (4.0%)		
Reimbursed as additional pay when unused	74 (11.9%)		
Requirements to access paid menstrual leave (*N* = 622)			
A once‐off doctor's approval	451 (72.5%)		
A once‐off mandatory diagnosis	84 (13.5%)		
Doctor's approval every time leave is accessed	30 (4.8%)		
A mandatory diagnosis and doctor's approval	43 (6.9%)		
Neither a diagnosis or doctor's approval	220 (35.4%)		
Frequency of leave (*N* = 623)			
Annual basis	**193 (31%)**		
Less than 12 days per year	18 (9.3%)		
12 days per year	85 (44.0%)		
More than 12 days per year	62 (32.0%)		
Other	28 (14.5%)		
Monthly basis	**430 (69.0%)**		
1 day	64 (14.9%)		
2 days	156 (36.3%)		
3 days	129 (30.0%)		
4 days	20 (4.7%)		
5 days	13 (3.0%)		
More than 5 days	7 (1.6%)		
Other	41 (9.5%)		
Need for workplace accommodations besides menstrual leave (*N* = 883, 3379 counts)			
Yes	**875 (99.1%)**		
Accessible and hygienic bathroom facilities, for example, sanitary disposal bins, hygienic hand washing facilities	758 (85.8%)		
The option to work from home	711 (80.5%)		
Flexibility to modify workstation, for example, use a heat pack, work in a more comfortable or private location	681 (77.1%)		
Flexible work hours and the ability to take unscheduled breaks as needed	599 (67.8%)		
Free or subsidised menstrual products available in workplace bathrooms	555 (62.9%)		
Other	75 (8.5%)		
No^a^	8 (0.9%)		
If paid menstrual leave had been available to you, would you have used it? (*N* = 73)^b^			
Yes	43 (59.0%)		
No	17 (23.3%)		
Unsure	13 (17.8%)		
If paid menstrual leave is currently available to you, would you use it? (*N* = 656)^b^			
Yes	475 (72.4%)		
No	89 (13.6%)		
Unsure	92 (14.0%)		
What do you think the leave should be called in Australia? (*N* = 614)			
Menstrual leave	450 (73.3%)		
Other (please specify)	164 (26.7%)		
Which of the following would you feel comfortable using when taking leave for menstruation?			
*Menstrual leave* (*N* = 575)			
Yes	489 (85.0%)		
No	86 (15.0%)		
*Period leave* (*N* = 508)			
Yes	303 (59.6%)		
No	205 (40.4%)		
*Women's leave* (*N* = 514)			
Yes	217 (42.2%)		
No	297 (57.8%)		
*Health leave (N* = 555)			
Yes	482 (86.8%)		
No	73 (13.2%)		
*Sick leave (N* = 515)			
Yes	375 (72.8%)		
No	140 (27.2%)		

^a^ As participants could select multiple options for this survey question, the numbers reported for this question are ‘counts’.

^b^ Participants who reported currently menstruating were asked the following question: ‘If paid menstrual leave is available to you, would you use it?’ Participants who reported no longer menstruating were asked the following question: ‘If paid menstrual leave had been available to you, would you have used it?’.

When subsequently asked how menstrual leave should be offered (paid, unpaid or reimbursed as additional pay when unused), most participants (84%) preferred menstrual leave to be paid. Most participants also preferred menstrual leave to require a once‐off doctor's approval (73%), be available monthly (70%) and be called menstrual leave (73%; Table 2). Besides menstrual leave, 99% of participants wanted other accommodations, with the most common being accessible and hygienic bathroom facilities (85.8%) and the option to work from home (80.5%).

Younger age significantly predicted support for paid menstrual leave (*B* = −0.06, Wald *χ* ^2^ (1) = 14.12, *p* = 0.000), with every one‐unit increase in age decreasing the odds of support by 95%. History of missing work also significantly predicted support for paid menstrual leave (*B* = 1.60, Wald *χ* ^2^ [1] = 3.57, *p* = 0.000), with every one‐unit increase in the history of missing work increasing the odds of support by 496%. This statistically significant model explained 16% of the variance (Nagelkerke's *R* ^2^ = 0.156).

The content analysis of an open‐ended question about potential positive impacts of paid menstrual leave on employees indicated that 95.8% of participants reported potential positive impacts, totalling 1210 counts (15 categories and 27 subcategories; Table 3). The most common categories were ‘*Better workplaces*’ and ‘*Productivity and improved work outcomes*’, with each category reported by 25% of participants. Participants also noted that the leave ‘*Promotes wellbeing*’ (19.6%).

**TABLE 3:** Content analysis of potential positive impacts of paid menstrual leave on employees (*N* = 672, total counts = 1210).

Categories (and subcategories)^a^	Number of participants^b^	Number of counts	Direct quote examples
**Better workplaces** Work environment and culture Morale Work/Job satisfaction Motivation Safety Relationships Trust and appreciation Loyalty Sense of belonging	168 (25.0%)	**189** 65 (34.4%) 36 (19.1%) 28 (14.8%) 17 (9.0%) 11 (5.8%) 10 (5.3%) 10 (5.3%) 10 (5.3%) 2 (1.1%)	‘Just the recognition of periods as real impact of people's lives alone would result in beneficial positive workplace culture’ ‘Having flexibility and support in how we work (and how/from where) can contribute to greater work satisfaction, morale and motivation/efficiency at work’ ‘Creating a safe and engaging workplace environment that takes into consideration the needs of employees—resulting in higher engagement and satisfaction and, by extension’
**Productivity and improved work outcomes** Productivity Quality of work Less mistakes and incidents Planning and scheduling	168 (25.0%)	**182** 150 (82.4%) 23 (12.6%) 5 (2.8%) 4 (2.2%)	‘Employers are getting better quality work for the days employees are present, with fewer mistakes made because employees are dealing with pain instead of focusing on work’ ‘Could help with planning—employers won't have people taking unplanned or unexpected leave anymore’
**Promotes well‐being**	132 (19.6%)	**164**	‘Encouraging greater well‐being among women. Helping women feel that their health and wellbeing is important’
**Feeling valued, cared, respected and understood**	78 (11.6%)	**92**	‘Employees feeling valued and understood within their workplace’
**Not having to work in pain and feeling comfortable** Comfort Not having to work in pain Working around others in pain	80 (11.9%)	**90** 47 (52.2%) 40 (44.4%) 3 (3.3%)	‘… means that we don't have to make things worse by being at work when experiencing debilitating pain’ ‘Employees will be able to stay at home if in menstrual pain and ones at work can continue without worrying about that person’
**Destigmatisation and recognition of menstruation**	56 (8.3%)	**70**	‘Recognition that menstrual pain is legitimate and like maternity leave, a need for women to have access to additional leave should they need it’
**Happiness**	51 (7.6%)	**63**	‘Much happier employee knowing they are cared for, knowing they can allow their bodies to rest when it asks for it, feeling more energetic after coming back from menstrual leave, not burning self out and therefore feeling more physically and mentally positive’
**Reduced stress and pressure**	61 (9.1%)	**61**	‘Less stress put upon women to “push through” and put on a happy face when they are in pain’
**Rest and recovery** Time to rest and recover Being able to care for yourself Burnout	52 (7.7%)	**56** 42 (75%) 10 (17.9%) 4 (7.1%)	‘Employees getting the time they need to look after their bodies’ ‘Knowing they have paid days off will allow them to not worry about missing a day or pushing through pain and then becoming burnt out’
**Job and financial security**	43 (6.4%)	**53**	‘It would mean so much to millions of women in the workforce and would be a massive step in women's rights and equality. We want to work as hard as we can, but it is hard to do that 100% when experiencing menstrual pain. The money would really help in supporting Australian families, couples and singles to survive with these ever‐growing cost of living rises’
**Improved use of leave (sick, personal etc.)** Better management of leave Not having to lie/justify of cause of sick day Predictability/planned leave Unexplained sick days Leave for those who don't normally have sick leave etc.	42 (6.3%)	**45** 30 (66.7%) 8 (17.8%) 3 (6.7%) 3 (6.7%) 1 (2.2%)	‘No need to use up annual leave or flexi time when unwell because of periods’ ‘Less random sick days’ ‘Women won't lie about why they need to take time off’ ‘Leave available for employees for wouldn't normally have leave to take, such as casual employees or staff on certain agreements excluding type of leave. Leave balances able to be utilised for their intended purposes’
**Equity and inclusion**	32 (4.8%)	**41**	‘More inclusive across the board, might get workplaces to think more about disability accommodations and gender equity’ ‘Realisation that sex differences exist and welcoming that extra education’
**Staff retention** Retention Staff turnover Absenteeism and presenteeism	36 (5.4%)	**38** 28 (73.7%) 5 (13.2%) 5 (13.2%)	‘Less staff turnover’ ‘Imagine the drop in absenteeism that women would already be taking if their menstrual pain is bad enough to take leave without pay’ ‘There would be less “presenteeism” as workers would take the time off’
**Dignity and empowerment**	28 (4.2%)	**38**	‘Capacity to meet health needs, manage symptoms and avoid diminished presentation at work (i.e., attending when lethargic or in pain and seeming tired/dishevelled)’ ‘Dignity (e.g., having to work knowing your clothes are blood stained)’
**No positive impacts**	28 (4.2%)	**28**	‘None, menstruation is a natural process, not an illness’

^a^ Categories are in bold and subcategories, where they were generated, are indicated with dot points.

^b^ All 672 participants who engaged in this section of the survey provided a textual response to this question. Responses that could be categorised in more than one category were allocated to each relevant category. For ease of presentation, only categories endorsed by 4% or more of participants are reported; therefore, seven categories and one subcategory are not reported in the above table.

In response to another open‐ended question concerning potential negative and unintended impacts of paid menstrual leave, 81.8% reported potential negative or unintended impacts (9.7%, reported no negative impacts, 8.5% left the question blank), totally 779 counts (11 categories and 16 subcategories; Table 4). The most common categories were ‘*Discrimination*’ and ‘*Strain on workplace functioning and productivity*’, which accounted for 18.4% and 16.3% of participants, respectively. Participants also noted ‘Negative attitudes and treatment from the workplace’ (14.1%) and ‘Potential abuse of leave’ (10.6%) as other potential negative or unintended impacts of paid menstrual leave.

**TABLE 4:** Content analysis of negative and unintended impacts of paid menstrual leave on employees (*N* = 672, total counts = 779).

Categories^a^	Number of participants^b^	Number of counts	Direct quote examples
**Discrimination** Employment discrimination General discrimination Reduced career advancement	130 (18.4%)	**145** 78 (53.8%) 44 (30.3%) 23 (15.9%)	‘… it could contribute to people who menstruate not being employed at the same rate as those that don't, so employers can avoid paying paid menstrual leave …’ ‘… may lead to women (and others who menstruate) not being given career progression or job opportunities as it is assumed they will take extra time off (much like how women are passed over for opportunities as it is assumed they will take maternity leave)’
**Strain on workplace functioning and productivity** Workplace productivity Segregation and conflict among co‐workers	109 (16.2%)	**118** 95 (80.5%) 23 (19.5%)	‘Role strain and increased rates of burn out among other employees covering workload’ ‘Disharmony among male vs female colleagues (or those who menstruate vs those who don't/post menopause)’
**Negative attitudes and treatment from workplace** Bullying Resentment Judgement Jealousy Backlash and harassment	95 (14.1%)	**114** 33 (28%) 32 (27.1%) 25 (21.2%) 14 (11.9%) 10 (8.5%)	‘Bullying or shaming from bosses or co‐workers’ ‘There could be some harboured resentment between the employees that take the leave and those that don't …’ ‘Jealousy from colleagues who don't get the menstrual leave’ ‘Might face backlash if you take it’ ‘Harassment from the employer‘
**Potential abuse of leave** Abuse of leave Being accused of faking need for leave	71 (10.6%)	**80** 71 (88.8%) 9 (11.3%)	‘There may be some people who do take advantage of this and that ruins it for others’ ‘Seen as accessing leave that others can't, not being believed that period pain is bad enough to need a sick day’
**No negative impacts**	65 (9.7%)	**71**	‘None, menstrual accommodation is a basic human right and does not negatively affect anyone’
**Increased stigma** Stigma Reinforces stereotypes	61 (9.08%)	**70** 50 (71.4%) 20 (28.6%)	‘Stigma surrounding actually using the leave you are entitled to. May become a badge of honour to not use the leave’ ‘It can reinforce the sexist stereotype that people on their periods are unable to work, emotional and essentially incompetent’
**Participants' response was blank**	57 (8.5%)	—	—
**Men and misogyny**	51 (7.6%)	**51**	‘Male employees may feel they “work harder” than women who access this leave which may further entrench misogyny within the workplace’
**Violation of privacy** Invasion of privacy Difficulty asking for leave	47 (7.0%)	**51** 44 (86.3%) 7 (13.7%)	‘The workplace will know the exact reason as to why you cannot come in and it may feel a bit invasive if they have to ask questions regarding the time off’ ‘… may be uncomfortable asking a non‐menstruating person for the leave as they might not understand’
**Unfairness and inequality**	42 (6.3%)	**44** (100%)	‘Unfairness across the board for all sexes. What will men get in return to even the playing field?’
**Feelings of guilt and shame**	32 (4.8%)	**35** (100%)	‘Feeling ‘guilty’/letting the team down—particularly as non‐menstruating co‐workers don't have the same leave’

^a^ Categories are in bold and subcategories, where they were generated, are indicated with dot points.

^b^ 672 participants engaged in this section of the survey, but only 615 responded to this specific question. Responses that could be categorised in more than one category were allocated to each relevant category. For ease of presentation, only categories endorsed by 4% or more of participants are reported; therefore, 11 categories are not reported in the above table.

## 4 Discussion

Our findings suggest support for paid menstrual leave among our sample, almost double that noted in previous Australian research [16]. Our results also demonstrate preferences for the name of the leave, how frequently it should be available and the mechanisms for accessing it. As such, our work provides valuable information for future legislative and workforce policy design and implementation.

Overall, 85% of participants were *Somewhat* to *Definitely* in favour of menstrual leave, and many also reported that they would use the leave if it were available. Support for paid menstrual leave was confirmed when the respondents were subsequently given other leave options such as unpaid leave or reimbursement in the form of additional pay for unused menstrual leave. These findings complement those of previous surveys by the Australian Council of Trade Unions with female union members and the Victorian Women's Trust with women currently menstruating or experiencing menopause, which found 74% and 84% support, respectively [17, 18]. These findings of preference for paid menstrual leave accord with the Victorian Women's Trust 2018 survey [19], where 80% of their participants supported this form of leave. At the same time, cost and financial impact of leave on employers and employees are common concerns expressed in the literature about menstrual leave [20, 21]. Spain has recently announced that its social security system will cover the cost of its new menstrual leave legislation [22], while in Japan and Indonesia, the leave is at employers' expense [21, 22].

In addition to employment leave, many participants desired other menstruation‐related workplace accommodations, the most common being accessible and hygienic bathroom facilities, the option to work from home and the ability to modify workstations. Such findings align with a 2021 survey from the Australian Department of Health [16] that demonstrated high support for employees to have access to free or subsidised menstrual products at work and flexible work arrangements, as well as the Victorian Women's Trust menstrual policy [19] that allows employees to work from home or in the office in a more quiet and comfortable area.

Our participants predominantly preferred a once‐off doctor's approval to access menstrual leave, consistent with countries like Spain and Italy [23]. However, 35% of participants preferred no requirements to access the leave, like the approach in Japan [21]. While there is no current research examining the impact of access requirements on leave utilisation, this is a strong concern for countries looking to develop menstrual leave legislation. For example, in Spain, there are concerns about the cost of obtaining a medical certificate, a requirement in that country's menstrual leave legislation, as it may outweigh the benefits for those in low‐paying jobs [24].

Younger age was a significant predictor of support for paid menstrual leave, suggesting there may be generational differences in the attitudes towards accommodations for menstruation [4, 25, 26]. A history of missing work for menstrual‐related pain was also a significant predictor of support for paid menstrual leave. From this finding and those from other studies, many women exhaust their sick and personal leave for menstrual‐related symptoms and often take unpaid leave or unpaid days off work [5, 6]. Self‐evidently, they would favour dedicated menstrual leave to protect their job and financial security. In our study, employment status, menstrual status and experience of debilitating pain were not significant predictors of support for paid menstrual leave and understanding the reasons for these findings may be made clearer with qualitative research.

The content analysis of written comments suggests that participants perceived more positive than negative or unintended impacts of paid menstrual leave. The most prominent category of positive impacts was ‘*Better workplaces*’. Participants expressed how workplace recognition of menstruation can foster a positive workplace culture that promotes safety and healthy relationships, which has been reported by women in Australian workplaces that have implemented menstrual leave, such as ModiBodi [26].

In contrast, ‘*Discrimination*’ was the most common category among the negative or unintended impacts of paid menstrual leave. Participants expressed concerns that paid menstrual leave may lead to employment discrimination, resulting in fewer employment and career advancement opportunities for women compared to their male counterparts. Numerous participants drew parallels with their experiences of workplace discrimination related to maternity and parental leave, which is echoed in reports highlighting gender‐based backlash to such leave policies [27]. Similar concerns were also expressed in the Australian Department of Health Menstrual Health Survey, which highlighted that the leave and ‘special treatment’ can perpetuate the idea that women are weak and, thus, provide a reason for discrimination [16]. Historical examples, such as Russia's 1927 removal of menstrual leave due to discriminatory practices, underscore the importance of addressing these concerns in policy development to ensure they promote ‘*Better workplaces*’ and prevent ‘*Discrimination*’ [23].

‘*Productivity*’ was a common finding in the analyses of open‐ended questions about the potential positive and negative or unintended impacts, which was also found in an American thematic analysis on paid menstrual leave [4]. Positive impacts on productivity included better quality of work, fewer mistakes and the ability to plan and schedule. Participants reported that they often struggle to perform at their full potential while menstruating in the workplace and that rest and recovery and looking after their wellbeing would lead to better quality work. In contrast, potential negative or unintended impacts of menstrual leave included strain on workplace functioning and productivity, negative attitudes and treatment from workplaces and the potential abuse of leave.

A strength of our study is its robust sample of 929 participants, allowing for diverse opinions and increased statistical power compared to similar Australian research with 500 participants [16]. Limitations include a sampling frame of women from social media and online support groups for menstruation‐related health conditions, who may be more inclined to support the leave. The participants were predominantly ethnically Australian identifying, highly educated, aged 35 and below and likely to have experienced debilitating pain or have a diagnosis for a related health condition. Also, we do not know whether our sample contained Aboriginal or Torres Strait Islander peoples, or our participants' parity or residential location (i.e., urban or rural). Future research should recruit under‐represented, culturally and socially diverse populations. More attention might be paid to understanding the perspectives of women in different residential locations and employment sectors, including those in manual and traditionally male‐dominated employment with environmental stressors (i.e., access to bathrooms). Further research is needed to develop an inclusive understanding of menstrual leave and to broaden perspectives by investigating the attitudes of a wider range of stakeholders such as men, colleagues, employers, employment lawyers and economists.

## Conflicts of Interest

The authors declare no conflicts of interest.
